# Exosomes: Potential in Cancer Diagnosis and Therapy

**DOI:** 10.3390/medicines2040310

**Published:** 2015-11-02

**Authors:** Phillip Munson, Arti Shukla

**Affiliations:** Department of Pathology and Laboratory Medicine, University of Vermont, College of Medicine, Burlington, VT 05405, USA; E-Mail: pmunson@uvm.edu

**Keywords:** exosomes, cancer, therapy, biomarkers, tumor microenvironment, pre-metastatic niche

## Abstract

Exosomes are membrane-bound, intercellular communication shuttles that are defined by their endocytic origin and size range of 30–140 nm. Secreted by nearly all mammalian cell types and present in myriad bodily fluids, exosomes confer messages between cells, proximal and distal, by transporting biofunctional cargo in the form of proteins, nucleic acids, and lipids. They play a vital role in cellular signaling in both normal physiology and disease states, particularly cancer. Exosomes are powerful progenitors in altering target cell phenotypes, particularly in tumorigenesis and cancer progression, with the ability to alter tumor microenvironments and to assist in establishing the pre-metastatic niche. Many aspects of exosomes present them as novel means to identify cancer biomarkers for early detection and therapeutic targets, and using intrinsic and engineered characteristics of exosomes as therapeutic devices to ameliorate the progression of the disease. This review outlines some of the recent and major findings with regard to exosomes in cancer, and their utilization as therapeutic tools.

## 1. Introduction

The ability for cells to communicate is quintessential to the biology of multicellular organisms. Intercellular signaling events are accomplished in many ways and depend on a complex array of networks and processes including direct contact, electrical and chemical components, soluble molecular messengers, or the secretion of membrane-bound vesicles. An emerging field in biological research focuses on exosomes as a seminal conduit for this cellular crosstalk. Exosomes were first described in 1981 as “exfoliations” from neoplastic cell culture monolayers [1], and have since gained significant momentum for their biological and therapeutic relevance. Nearly all mammalian cell types have been shown to produce exosomes and their presence has been confirmed in many bodily fluids, including urine, blood, saliva, and amniotic fluid [2]. 

**Figure 1:**
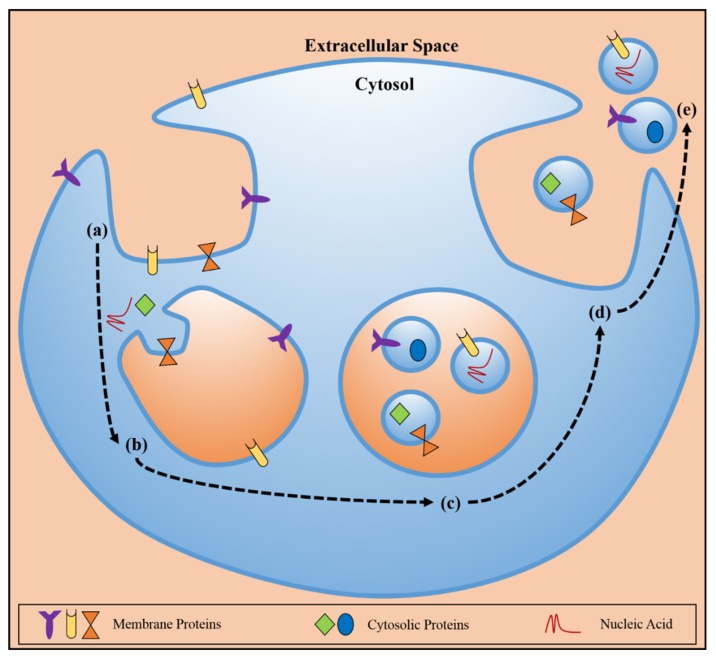
Schematic representation of exosome biogenesis and release, with depictions of membrane proteins, cytosolic proteins, and nucleic acid. (**a**) Endocytosis at the plasma membrane leads to the (**b**) immature endosome where invagination of the endosomal membrane occurs and molecular cargo is loaded into the newly forming particles. (**c**) The mature endosome, or multivesicular body (MVB) contains the exosomes and (**d**) upon fusion of the endosomal membrane with the plasma membrane, (**e**) the exosomes are released to the extracellular environment, maintaining the producer cell membrane topology and housing protein/nucleic acid cargo, which then can travel to recipient cells, locally or distally, communicate molecular information, and/or induce phenotypic changes.

Exosomes are small, 30–140 nm, membrane-bound particles defined by their origin from the endosomal pathway (Figure 1), and are not to be confused with microvesicles which are larger (~1000 nm) and are shed directly from the plasma membrane [3]. The content of exosomes is another pivotal feature of their classification and ability to carry information and cargo, as they are enriched in RNA species (*i.e*. mRNA, miRNA), proteins, biofunctional lipids, and occasionally DNA [4]. With communication at the forefront of their function, exosomes can also participate in waste removal, antigen presentation, and the induction of pro-inflammatory cytokine release [5].

Interestingly, exosomes are attributed to playing roles in both normal physiological conditions (immune surveillance, neural plasticity, tissue repair, stem cell maintenance, and blood coagulation pathways) as well as in the pathological processes of many disease states [6]. For this review, we will focus on the role of exosomes in cancer, although they are associated with the pathogenesis of viruses like HIV-1, the progression of Alzheimer’s and Parkinson’s diseases, the spread of prion proteins, and inflammatory conditions [7]. The roles of exosomes in disease demonstrate their prospective utilization as either therapeutic targets or, potentially, as therapeutic agents.

Production of exosomes occurs at the early endosome, resulting in the formation of a multivesicular body (MVB), however, the exact mechanisms of their biogenesis is not well understood. The early endosome is the direct product of a primary endocytic event at the plasma membrane. Invagination of the endosomal surface and subsequent pinching off of the membrane creates the exosomes, also referred to at this stage as intra-luminary vesicles.

Two major pathways are suggested in the production of exosomes at the endosomal membrane: the Endosomal Sorting Complex Required for Transport (ESCRT)-dependent pathway and the ESCRT-independent pathway. The ESCRT-dependent pathway requires an accessory protein, ALIX, and is comprised of four complexes: ESCRT-0, which identifies and loads ubiquinated proteins on the endosomal surface; ESCRT-I and ESCRT-II, which cause membrane budding; and ESCRT-III, which is involved in membrane separation. The ESCRT-independent pathway is proposed to involve lipids, such as sphingosine-1-phosphate and ceramide, microdomains enriched with tetraspanins, and the enzyme sphingomyelinase [8]. The study by Colombo *et al.* illustrated that disrupting certain parts of the ESCRT machinery results in decreased production of exosomes *in vitro* [8]. Recent evidence reports an exosomal production pathway requiring the membrane protein syndecan and cytosolic protein syntenin, in which these two proteins interact with the ESCRT-accessory component ALIX, the GTPase ADP Ribosylation Factor 6 (ARF6), proteolipid protein D2, and the endoglycosidase heparinase [9]. In conjunction with these pathways, it should be noted that there are four major requirements for exosome biogenesis: cytoskeletal components such as actin and microtubules; molecular motors such as kinesin and myosin; molecular switches which are primarily small GTPases; fusion machinery, and tethering factors such as SNAREs [10].

The exosomal membrane reflects aspects of the endosomal membrane composition, and maintains the same membrane topology as the plasma membrane of the parent cell. The exosomal membrane, therefore, is enriched in MVB-related proteins such as flotillins, Annexins, GTPases, Rab, and SNAREs; proteins involved in MVB biogenesis such as ALIX, Tsg101; and membrane-microdomain associated proteins, particularly certain tetraspanins (CD9, CD63, CD81, and CD82) [3]. The lipid composition of exosomes is enriched in sphingomyelin, cholesterol, and ceramide. Moreover, the membrane of exosomes can also present Major Histocompatibility Complex (MHC I/II) molecules and/or antigens, depending on the cell type from which the exosome was secreted. These specific proteins and lipid molecules are important tools in the classification of exosomes and are attractive targets for the identification of novel biomarkers [11]. 

The internal cargo of exosomes is noticeably dissimilar to that of the producer cell’s cytoplasmic content, indicating that cargo loading into exosomes is not a simple, diffusive, or unregulated process. This selective packaging of certain proteins and RNA species into exosomes adds another layer of complexity to understanding their biogenesis and indicates a sophisticated sorting process. Only some elucidations have been made as to the relationship between certain biogenesis/sorting molecules and their respective cargo, such as ESCRT-0 loading ubiquinated proteins. ESCRT-II has been shown to specifically bind mRNAs, suggesting its role in the cargo sorting of mRNA into exosomes [12]. 

Proteins and RNA species identified in exosomes so far have been deposited in the readily available online database, ExoCarta [13]. The most commonly identified exosomal proteins are heat shock protein (HSP)-8 and CD63. Cytoskeletal proteins are commonly identified (β-actin, cofillin, moesin, and tubulins) in exosomes, as well as proteins involved in cellular signaling pathways (β-catenin, WNT5B, and Notch ligand Delta-like 4) [14]. Due to the fact that cargo recruitment is not well understood, it can only be postulated that specific chaperone proteins found in exosomes are truly regulators of the process, like HSC, HSP90, 14-3-3, and PKM2 [15]. Discerning the function of these proteins presents a challenge as other proteins are incorporated based on their interactions with lipid-raft associated molecules which become incorporated into the MVB [16].

Notably, one of the more interesting components of exosome cargo is their enriched population of small non-coding RNAs, specifically microRNA (miRNA). Other RNAs are also incorporated, such as piRNA, snoRNA, scaRNA, Y RNA, siRNA, tRNA fragments, and vault RNA [17]. Nearly half of the genes in our cells are regulated by miRNA [18], further demonstrating the signaling capacity and modulatory capabilities of exosomes on target cells.

Exosomes are released to the extracellular space upon fusion of the MVB with the plasma membrane. This process is mediated by a subset small, vesicular transport regulation GTPases known as Rab27A, Rab11, and Rab31 [19], and another reported mechanism for secretion, specifically for exosomes bearing WNT, involves the SNARE protein YKT6 [20]. Alternatively, some exosomes are not released, and are instead destined for lysosomal degradation, which has been attributed to MVB lipid composition where it appears that MVBs with cholesterol-poor membranes, and/or have lysobisphosphatidic acid present are targeted for the lysosome [21].

Target cell specificity is not yet fully understood but is likely determined by adhesion-associated molecules present on the exosomal surface, such as integrins and SNAREs, with the possible influence of tetraspanins complexes [22]. There are several fates of exosomes once bound to a recipient cell, prompting what signaling information is delivered: the exosome can bind and associate with a membrane receptor or dissociate; direct fusion with the plasma membrane and unloading of cargo to the target cytosol; or endocytic internalization [3].

## 2. Exosomes and Cancer

**Figure 2:**
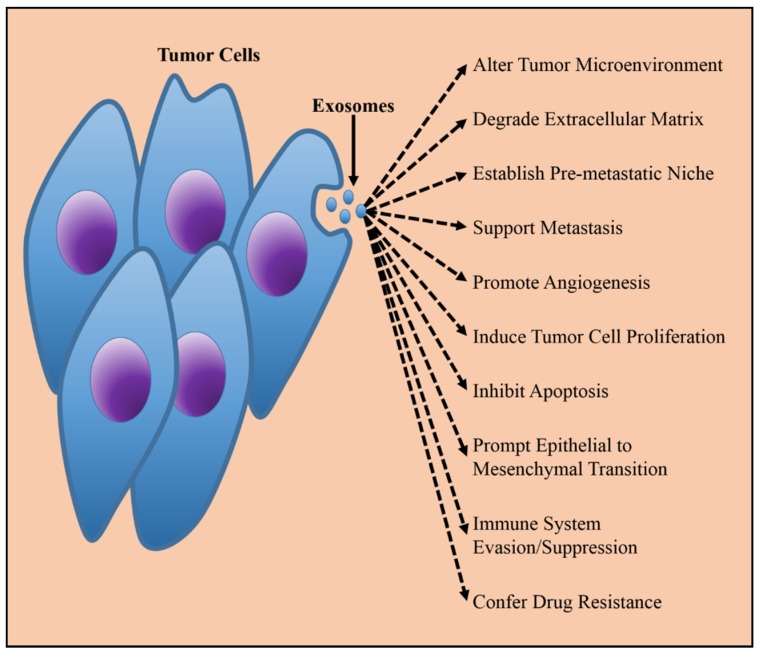
Tumor derived exosomes are released constitutively from cancer cells, and are capable of relaying information which reprogram target cells and modifies physiological environments in manners beneficial to cancerous growth and metastasis.

There is substantial and mounting evidence on the dynamic role of exosomes secreted by cancer cells in contributing to tumorigenesis, disease progression, metastasis, angiogenesis, extracellular matrix (ECM) remodeling, immune evasion, chemoresistance, and the establishment of the pre-metastatic niche [7] (Figure 2). Exosome secretion by tumor cells is markedly up-regulated, as is observed by increased exosome collection from cancer cell cultures or serum of cancer patients compared to non-cancerous conditions [23,24]. Tumor-derived exosomes are capable of exchanging information between neighboring cancer cells and, more notably, can communicate with distant sites and various cell types. The capability of tumor exosomes to house tumorigenic information and induce distal or local cellular responses that promote disease pathogenesis make tumor exosomes an attractive tool in identifying cancer biomarkers, uncovering molecular mechanisms to cancer biology, and exploiting exosomes for therapy.

Communication with the tumor microenvironment is vital for tumor progression and metastasis. Cancer cells secrete exosomes to reprogram their environs and establish favorable conditions for tumor growth and invasion of healthy tissues. This microenvironment is comprised of the ECM and stromal cells including fibroblasts, endothelial and inflammatory immune cells, and tumor-associated vasculature [25]. Evidence is also accruing that links adipose stromal cells, or adipocytes, to promoting the tumorigenic microenvironment, especially in obesity-related cancers [26]. Fibroblasts synthesize ECM and are essential to repaving this extracellular network during aberrant cell growth. Cancer exosomes can induce fibroblasts to become more activated in laying the framework for this favorable tumor microenvironment by eliciting the TGFβ/Smad pathway in target fibroblasts [27]. Fibroblast remodeling of the tumor microenvironment can also be promoted by the exosomal secretion of ECM metalloproteinases from tumor cells [28]. 

Epithelial-to-mesenchymal transition (EMT), a hallmark of tumor microenvironments becoming more aggressive and metastatic, can only be accomplished through intercellular communication and it has recently been reported that tumor exosomes are a contributing factor [29]. This process of EMT is led by oncogenic transmission that is possibly mediated by exosomal cargo transfer which modulates certain aspects of differentiation associated with tumor-driving EMT [30].

Brain tumor cells expressing an oncogenic epidermal growth factor receptor (EGFR) were shown to export and deliver this mutant EGFR to other cells, thus transferring oncogenic activity leading to activation of MAPK and Akt signaling pathways, morphological transformation, and anchorage-independent growth [31]. Such alterations can lead to consequent production of angiogenic factors such as vascular endothelial growth factor (VEGF) which can facilitate vascularization of the tumor mass. 

Cancer exosomes are clearly powerful mediators with the aptitude for changing the behavior of neighboring cells. This becomes even more evident with their ability to promote the formation of the pre-metastatic niche. For metastasis to occur, not only do cancer cells need to migrate to a new environment, but that environment must be conditioned appropriately to allow colonization. 

An elegant *in vitro* and *in vivo* experiment by Sung *et al.* [32] demonstrated that exosome secretion was required for directional cell movement and persistent migration of cancer cells. The experiments by Sung *et al.* utilized live-cell imaging to show that exosome secretion directly preceded and enabled adhesion assembly via an exosome induced autocrine signaling with fibronectin housed inside exosomes as the critical component. By inhibiting exosome biogenesis, the authors of this experiment showed that directional cell movement was defected and by rescuing the ability of cells to produce exosomes led to reestablished directional motility. Therefore, cancer exosomes are capable of secreting and delivering necessary ECM molecules to modulate integrin and adhesion formation to drive the migration and invasion of cancer cells.

It has been illustrated that exosomes derived from metastatic melanomas promoted metastatic behavior of primary tumors through the horizontal transfer of MET oncoprotein to bone marrow progenitor cells, a process referred to as “educating” for metastatic colonization [33]. 

Liver pre-metastatic niche formation was shown to be induced from pancreatic ductal adenocarcinomas (PDAC) derived exosomes that expressed high levels of macrophage migration inhibitory factor (MIF) and led to a fibrotic-microenvironment. Via a MIF-blockade, liver pre-metastatic niche formation was prevented. Measurement of exosomal MIF levels in patients with stage 1 PDAC revealed a correlation of high exosomal MIF levels with the development of liver metastasis as compared to patients with low exosomal MIF [34].

Intra-vital imaging of cancer exosome uptake by non-cancer cells using the Cre-LoxP system, showed that mRNA cargo delivered to non-malignant cells induced enhanced migratory potential and metastatic capacity [35]. 

In addition, miRNAs have an intriguing role in cancer and exosomes. MiRNAs are non-randomly added to exosomes and carry functional information from cancer cells which can phenotypically change target cells in a fashion that shapes and alters microenvironments to allow favorability for cancer cell growth and invasion [36]. Melo *et al.* showed that miRNA maturation occurred in exosomes after their incorporation into vesicles [37]. When compared to miRNA content of exosomes derived from healthy cells, the cancer exosomes had a disproportionately higher concentration of mature miRNAs. This suggests that cancer exosomes might not only act as simple postage boxes, but are rather active facilitators in the processing of their own cargo. Breast cancer exosomes with functional miRNAs are capable of altering target cell transcriptomes and instigating non-cancer cells to become more tumorigenic [37]. The miRNA family, miR-200, regulates the process of EMT, mentioned above, and was seen to be increased in serum exosomes of cancer patients [38].

A recent study by Valenzuela *et al.* [39] presented that a series of tumor cell lines all secreted exosomes containing the inhibitors of apoptosis (IAPs) Survivin, cIAP1, cIAP2, and XIAP. The authors suggested that cancer exosomes contain these IAPs as a possible warning signal or as an added layer of protection to the rogue proliferating cells from an ever-changing tumor microenvironment [39].

The effects of cancer exosomes on the immune system is two-handed, as they can induce immunosuppressive functions that uphold tumorigenesis or can provide a boost to the immune response to tumors. Apoptosis of CD8+ T cells can be induced by cancer exosomes through the death receptor pathway [40]. Cancer exosomes can lead to further T cell dysregulation by inducing the proliferation of regulatory T cells while inhibiting effector T cell proliferation [41]. Additionally, cancer exosomes can negate the cytotoxic functions of natural killer cells [42].

On the other hand, cancer exosomes can spread antigens, increasing dendritic cell presentation of those antigens. Also, exosomes can interact with memory T cells leading to antigen-specific immune responses against the tumors [43].

Cancer exosomes are also implicated in tumor resistance to chemotherapeutic drugs. The removal of cisplatin and trastuzamab from cancer cells by exosomes indicates a drug-scavenging function [40]. It was also shown that certain chemoresistant cancer cells could horizontally transmit their drug-resistant phenotypes through their exosomal miRNAs [44], and an increasing number of studies are linking exosomal miRNAs to the ability of cancer cells to acquire drug resistance and conduct that resistance to other cancer cells [45]. Mesenchymal stem cells (MSC) are known to be involved in chemotherapeutic drug resistance and MSC-exosomes have been implicated in promoting drug resistance in gastric cancer by activating the calcium/calmodulin-dependent protein kinase (CaM-Ks) and Raf/MEK/ERK kinase cascade [46].

A recent review by Braicu *et al.* outlines even further how secreted messages from cancer-derived exosomes use both membrane and cytosolic constituents, particularly miRNAs, to act as critical components of the tumorigenic circuit that disrupts the normal condition of healthy cells into the development of oncogenesis [47]. 

## 3. Exosomes in Therapy

Unsurprisingly, due to their strong implications in cancer pathogenesis and biological compatibilities (*i.e*. their ability to cross physiological barriers like the blood–brain barrier), exosomes are strong candidates for myriad therapeutic applications. These possibilities include targeting exosomes that appear to be progenitors in cancer progression, engineering exosomes as therapeutic devices, and discovering novel biomarkers for early diagnosis and identifying molecular targets. Aside from cancer, beneficial effects of therapeutic exosomes have already shown promise in myocardial ischemia reperfusion and kidney injury [48], myocardial infarctions [49], muscle or bone regeneration [50], arthritis [51], nerve regeneration [52], multiple sclerosis [53], and neurodegenerative diseases, such as Alzeihmer’s or Parkinson’s [54].

**Figure 3:**
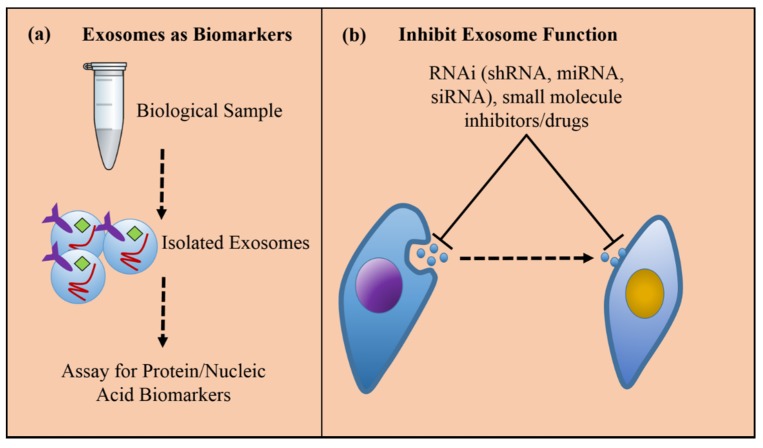
(**a**) Exosomes can be isolated from cell culture supernatants or patients’ bio-fluids to assign diagnostic and prognostic signatures of cancer by profiling exosomal proteins or RNAs; therefore, exosomes potentiate a non-invasive, or liquid biopsy, technique for assessing tumorigenesis and cancer progression. (**b**) Inhibiting exosome function is one particular therapeutic strategy for pacifying the cancer promoting effects of tumor-derived exosomes either by blocking the formation and release of the exosomes from the producer cell, or preventing uptake of the exosomes in the target cell.

Due to their selective cargo loading and resemblance to their producer cells, exosomes are valuable for discovering cancer biomarkers (Figure 3). With increasingly improving isolation techniques from cell culture and patient blood, and methodology for characterizing cancer exosome components, scientists are utilizing exosomes to identify molecules to target cancer more effectively and apply more personalized techniques to detection, diagnosis, and prognosis. Protein characterization by mass spectrometry [11], as well as immunocapture techniques for identifying and quantifying peptide and nucleic acid (miRNA, mRNA, etc. *etc.*) profiles [55] and commercially-available products already provide useful approaches to biomarker discovery. Some of the most recent cancer exosome biomarker studies include complete proteome analysis of melanoma exosomes [56] and circulating biomarkers [57], miRNA biomarker analysis of esophageal adenocarcinoma [58], prostate cancer [59], glioblastoma [60], serum miRNAs for acute myeloid leukemia [61], colorectal cancer [62], gastric cancer [63], urinary exosomal miRNAs for ovarian cancer [64,65], pancreatic cancer specific proteoglycan [66], proteomic biomarker profiling of cholangiocarcinoma [67], non-small cell lung cancer [68], glioma [69], and salivary exosomes for oral cancer [70]. In addition, it has been discovered that circular RNAs (circRNA) are stably expressed in exosomes and these circRNAs are suggested to be a promising candidate for biomarkers in cancer [71]. These examples provide insight that exosomes can be used as a more sensitive and less invasive technique to cancer diagnostics.

Very recently, the cell surface proteoglycan, glypican-1, was identified as being specifically enriched on cancer exosomes. Monitoring glypican-1 on circulating exosomes demonstrated specificity and sensitivity in distinguishing between healthy subjects and patients with benign pancreatic cancer from early/late stage pancreatic cancer patients [66]. Glypican-1 on circulating exosomes may be an efficient non-invasive screening tool for pancreatic cancer, and exemplifies the possibilities of exosomes for cancer diagnostics.

Attenuating the production and release of exosomes from tumor cells is one important therapeutic paradigm given that circulating exosomes nearly double in cancer patients and their cargo promote tumor progression and spread (Figure 3).

One such method would be to inhibit certain molecules that are required for exosome formation within the cell (i.e. the endosomal pathway), such as ceramide synthesis via the sphingomyelinase pathway. The use of amiloride to reduce exosome production and reduce tumor progression was observed *in vivo* via myeloid-derived suppressor cells which suppress T cell activation [72], but similar results were not seen with amiloride treatment of prostate cancer cells [73] suggesting that this mode of inhibition is cell-type dependent. Other factors that are involved in exosome biogenesis, such as the ESCRT pathway, and the syndecan proteoglycan and adaptor syntenin are possible targets also. 

The application of RNAi to inhibit certain gene regulation is of particular interest as their mechanics are becoming better understood, and the design of functional small interfering RNAs (siRNA) is improving to the point of preclinical and clinical trials [74]; for example, targeting the GTPase RAB27a which is required for the release of some tumor exosomes. RNAi and small molecule inhibition of targeted exosome biogenesis molecules can effectively knock down certain production characteristics of exosomes and be utilized for preventing for preventing exosome dissemination from diseased cells which might in turn lead to spread of disease phenotypes to target cells. The mechanisms for this action are either by gene knockdown by RNAi, such as engineered shRNAs that bind to, and prevent translation of exosome-production machinery including ESCRT proteins and/or GTPases involved in producing exosomes [75]. Peinado *et al*. demonstrated in their experiments mentioned above that RNAi of Rab27A GTPase in melanoma cells greatly abrogated exosome production and bone marrow education, consequently reducing the metastatic potential of the cancer [33]. Another study in mammary carcinoma cells led to decreased primary tumor growth and lung dissemination upon a blockade of RAB27a [76]. In addition, other GTPases that serve as factors in the docking/fusion of the MVB to the plasma membrane, can serve as potential targets for deregulating exosome secretion from tumor cells. 

Sung *et al*. illustrated in their experiments that knockdown of Rab27a and Syt7 reduced cancer exosome secretion between 2.2 and three-fold fewer compared to normal cells and also dysregulated cell polarization and migratory persistence [32].

Another possible target for inhibiting the tumorigenic function of cancer exosomes is to prevent the fusion or uptake of exosomes by target cells. One experiment prevented tumor-derived exosome uptake by cells through blocking phosphatidylserine with diannexin [77].

It should be noted, however, that this mode of repealing exosome function poses potential complications in that many normal physiological processes might be inadvertently afflicted.

An evolving approach in therapeutic exosomes is using them as drug delivery devices. Exosomes are ideal vehicles for molecule delivery (proteins, RNAs, small molecule drugs/drug oligonucleotides, *etc.*), due to their biocompatibility, stability in circulation, and ability to target them to certain cell types. Small interfering RNAs (siRNA) have enormous potential as therapy with their gene-knockdown effects, but are difficult to employ due to their high instability. Exosomes provide an innovative and newly popular device for carrying siRNAs, as well as shRNAs, miRNAs, and mRNAs. The expression profile of tumor exosome miRNAs becomes dysregulated in many cancers and can be used for tumor characterization and diagnostics, as well as therapeutic payload [78]. One study exemplified this aspect by loading MSC-exosomes with miR-146b and managed to reduce primary brain tumor growth in rat glioma by intra-tumoral injection [79]. Elucidating the natural mechanisms of miRNA loading into exosomes is imperative to progressing the use of miRNA as therapeutic cargo.

**Figure 4:**
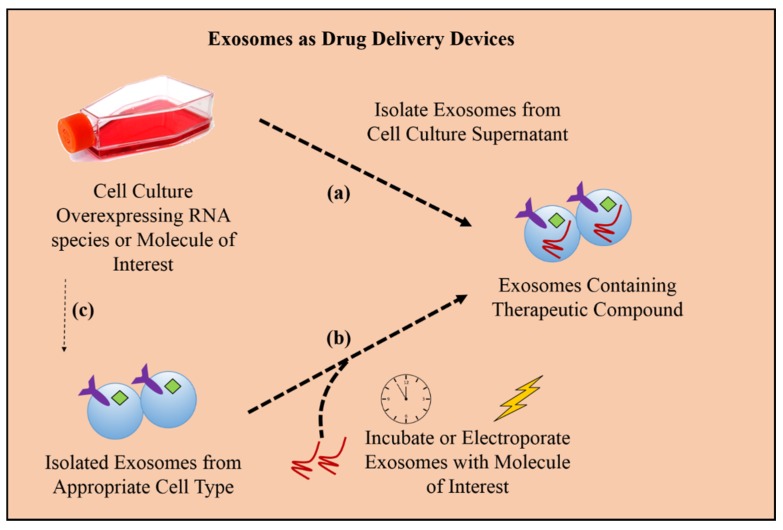
Loading exosomes with therapeutic cargo, such as RNA species for gene knockdown in targeted cancer cells or small molecule drugs of interest, can be achieved in two ways: (**a**) endogenously, by collecting exosomes from cells overexpressing the molecule of interest, or (**b**) exogenously, by collecting exosomes from an appropriate cell culture that produces exosomes suitable for specific targeting and then incubating or electroporating the exosomes with the molecule of interest. Once the exosomes are successfully loaded, they can be used for downstream therapeutic applications. (**c**) Additionally, it is theoretically possible to combine the two methodologies as a more comprehensive approach to loading with molecules into pre-engineered exosomes.

Drug loading can be accomplished either endogenously or exogenously (Figure 4). Endogenous, or passive, loading is carried out by overexpressing the RNA species or molecule of interest in producer cells. This passive loading is enabled by the cell’s native exosomal loading mechanisms and results in exosomes that contain the drug prior to isolation. Exogenous, or active, loading begins with exosome collection and requires either co-incubation or electroporation of the exosomes with the drug/molecule of interest [7]. Theoretically, it is possible that one could use exogenous drug loading on previously endogenously loaded or engineered exosomes as a more wide-ranging tactic to this methodology.

Additionally, it may be possible to use viral packaging strategies [80] to load exosomes with molecules [81] and marketed kits have become available to load exosomes in culture with proteins of interest, for example the XPack technology from System BioSciences [82].

Exosomes targeted to specific cell and tissue types can enhance specific uptake and reduce off-target deliveries. Cell or tissue targeting can be achieved by engineering exosomes to express plasmid fusion constructs with targeting ligands fused to extracellular membrane proteins. For example, exosomes were collected from immature dendritic mouse cells engineered to express Lamp2b fused to a tumor-targeting integrin and loaded with doxorubicin by electroporation. Intravenous injection of the engineered exosomes delivered doxorubicin specifically to the specific integrin-positive breast cancer cells leading to inhibition of tumor growth, whereas untargeted exosomes localized to the liver and spleen [83]. Different studies have shown that brain endothelial cell-derived exosomes are successful at crossing the blood–brain barrier to deliver anti-cancer drugs in a brain cancer zebrafish model [84] and that intra-tumoral injection of exosomes engineered to express an anti-tumor miRNA reduced glioma growth in rat models [79].

Choosing the correct cell line for therapeutic exosome production is important for a few reasons. The exosome must be lacking in immune-stimulating activity to prevent unwanted immune effects in target tissues. For this immunogenic reason, immature dendritic cells have been favorable choices [85]. Cell choice can also dictate the native population of exosomal surface proteins that might have a desirable ligand-receptor interaction with the proposed target cell. Finding this optimal producer-target cell combination is vital to producing exosomes for therapy. There is also the opportunity to generate exosomes with therapeutic cargo and ideal surface moieties using semi-synthetic processes for target cell specificity. Strategic advances are being made in producing exosomes with targeted peptides via glycosylation sites for enhanced targeted delivery of exosomes for therapeutics [86].

Tracking exosomes *in vivo* after injection is becoming more feasible in the literature and methodologies are being established using fluorescent labels or membrane dyes [33,81,87]. These technologies allow researchers to resolve the biodistribution and local enrichment of injected exosomes. Tracing the transfer of functional exosomal cargo, such as RNAs, within the tumor microenvironment *in vivo* can provide researchers with the identities of possible targeting sites for anti-cancer drugs and engineered exosomes [88].

Human MSC-derived exosomes, which have intrinsic therapeutic activity, appear to be promising producers of exosomes for therapeutic applications and drug delivery as they are known to have successful therapeutic benefits in diseased animal models and display immunosuppressive activity [89]. MSC-exosomes delivered to mouse breast cancer cells delivered molecules which led to the down-regulation of VEGF thereby decreasing tumor growth by suppressing angiogenesis [90].

The role of MSC-exosomes in promoting drug resistance in gastric cancer, as mentioned in the previous section, can be inhibited by blocking the CaM-Ks/Raf/MEK/ERK kinase cascade [46].

Exosomes have potential applications as cancer vaccines, as well. Exosomes loaded with α-galactosylcerimide and tumor-specific antigen can activate cancer-specific adaptive immune responses decreasing tumor growth [91] and, separately, isolated tumor-derived exosomes carrying tumor antigen were shown to effectively induce anti-tumor immune responses in primary and metastatic mouse melanoma models [92].

## 4. Conclusions

The idea of improving healthcare through personalized medicine is a growing field. Personalized medicine designates that tumor treatment be molded to the individual’s characteristics, biological signatures, and response to specific treatment. Hence, exosomes hold a spot in the development of efficacious personalized therapeutic techniques given their use for biomarker discovery and personalized diagnostic capacities. In the future, it might also be possible to isolate circulating exosomes from an individual, or from specifically harvested cell types, load them with specific molecules, *in vitro* with techniques mentioned above, tailored to a specific therapeutic strategy, and redeliver the modified exosomes back to a patient to induce a relevant response (*i.e.* reduce tumor growth).

Disease intervention with exosomes is an exciting new avenue in therapeutics with novel strategies for cancer treatment. There is promising evidence supporting the use of exosomes as diagnostic tools for discovering biomarkers, targeting exosomes to inhibit their disease-related functions, exploiting them as drug delivery devices, and utilizing their inherent therapeutic potentials. Further investigation is required to drive exosome-based therapeutics to the next level of research and eventual clinical trials that will clarify the complex aspects of exosomes that both promote and mollify malignant environments.
